# The Bistoury for 1870

**Published:** 1870-01

**Authors:** 


### The Bistoury.

ELMIRA, N. Y., JAN., 1870.

The BISTOURY is published Quarterly, upon the 1st of April, July, October and January, at Fifty Cents a year in advance.

Club Rates :--$25, will be paid for every 100 subscribers.

### THE BISTOURY FOR 1870.

With the present number the fifth year and fifth volume of the *Bistoury* closes.

When, five years ago, we commenced the publication of our journal,. making it free to all who desired it, we had no idea that it would grow into its present proportions and usefulness. We first issued 1200 copies. In a month the circulation was increased to nearly 3000 copies. In four years it had reached the enormous figure of 22,000, with orders still flooding upon us. It then became evident that we could not afford so large a circulation at our own expense, so that we resolved to make our journal a quarterly, issue it in magazine form, and see whether our subscribers would pay its way. Our friends predicted disaster—thought we could not possibly retain one-third of our free subscribers as paying ones. But we *have* done so, and far better, too; so that the BISTOURY is an established fact, and is looked upon as one of the necessities of every family that receives it. We have improved it with every number and intend further to beautify and enlarge it as our means will allow. We further promise our patrons that as soon as our circulation reaches 25,000 copies, for which we receive *pay*, that we will make it a monthly and at the *same price of subscription*.

No better articles have appeared in any magazine of the land than those contributed by Ben. H. Pratt and Ishmael. They have been universally read, copied, and admired. Our readers have become so attached to them that we cannot afford to lose their contributions. We have therefore secured their services as regular contributors to the next volume

In the main, the BISTOURY will be conducted in the future as it has been in the past. We will endeavour to instruct the people upon the laws of health and hygiene—give them useful hints and formulæ for the management of the sick, and expose all manner of quackery and humbuggery that is usually practised upon persons suffering from disease.

We will endeavour also, to render the BISTOURY valuable to the physician; will give him a synopsis of all that is transpiring in medicine and surgery, and condense, in a few pages, that which would require weeks to read in full. Our selections in "medical items and news" are taken from fourteen of the best medical journals of the world, costing us a subscription price of more than fifty dollars a year. All the information contained in these journals is condensed and placed before the physician in a comprehensive and available style, *for fifty cents*.

We hope our patrons will send in their subscription money for the new volume immediately, and that they will induce others to send with them. Just ask your neighbor to join you when you send your subscription for the new year, and so make the inclosure an even dollar

